# Effect of Speed Humps on Ambulance Delay

**DOI:** 10.7759/cureus.33722

**Published:** 2023-01-12

**Authors:** Erdoğan Öz, Osman Küçükkelepçe, Osman Kurt, Abuzer Cihat Çavuş

**Affiliations:** 1 Family Medicine, Adıyaman Provincial Health Directorate, Adıyaman, TUR; 2 Public Health, Adıyaman Provincial Health Directorate, Adıyaman, TUR; 3 Engineering, Adıyaman Provincial Health Directorate, Adıyaman, TUR

**Keywords:** speed hump, speed bump, speed breaker, ambulance response time, ambulance delay

## Abstract

Introduction: Speed humps allow vehicles to slow down their speeds, but they also cause emergency vehicles to waste time on their way to their destinations. The study aims to determine the delay times alone and queue delay time of ambulances passing through speed humps.

Methods: Three types of ambulances (Van, Multiple Victim Assistance, Bariatric) and vehicles (Truck, Lorry, Van) are passed in a controlled manner through speed humps at different speeds in three streets of Adıyaman province of Turkey. Ambulances and vehicles are slowed down to 15 km/h while passing the speed hump for safe passage. Passing and lost times were calculated with the help of a stopwatch (Catiga CG-503; Catiga Electronics Company, Hong Kong) and a global positioning system (GPS) speedometer (Vjoycar smart speedometer; Vjoy Car Electronics Limited, China). Differences in passing times in the absence and the presence of speed humps, determined with the speed equation formula (t=x / V), were lost timings or delay timings for ambulances and vehicles.

Results: In the first region, the lost time for the van ambulance with a speed of 70 km/h was 8.41 seconds, 10.14 seconds for the multiple victim assistance ambulances, and 9.56 seconds for the bariatric ambulance. While there was a truck in front of the van ambulance with a speed of 50 km/h, the lost time was also the queue delay time for the ambulance and was 54.96 seconds, with a lorry 42.81 seconds, and 7.02 seconds with a van. In the second region with a double-speed hump, the lost time for the van ambulance with a speed of 60 km/h was 9.94 seconds, 16.32 seconds for the multiple victim assistance ambulances, and 14.49 seconds for the bariatric ambulance. Ambulances did not waste time in the third region, as ambulances and other vehicles do not have to slow down.

Conclusion: Ambulances waste time by themselves or due to the vehicles in front of them passing speed hump. As the speed of ambulances increased, the lost time also increased. So, more time is lost when the ambulance needs to go faster.

## Introduction

The incidence of the population living in cities is increasing daily, and according to the prescience of the United Nations, 60% of the world's population will live in cities by 2030. So it is important to make roads safer for pedestrians, vehicles, and those waiting for emergency assistance [1-3].

In severe trauma patients, the inability of the ambulance to reach the case within 4 minutes reduces the likelihood of survival by 30% [4]. Each minute of delay in the ambulance response time increased the mortality risk by 8-17% in all emergencies [5]. In another study, each 1-minute reduction in ambulance response time increased the survival rate by 5.2% [6].

For safer traffic flow of pedestrians, speed breakers and traffic calming devices in the form of vertical elevations on the road pavement are used. These can be named speed hump, speed bump, speed table, and speed cushion/speed lump/speed pillow [7]. On the other hand, the speed hump and the speed bump are often used interchangeably in the literature [8]. The speed of safe passage of the vehicle varies according to the height, length, and shape of traffic calming devices [9].

In most studies, the speed hump is 7.62-10.16 cm in height and 3.66-4.27 meters in length; the speed bump is 7.62-15.24 cm in height and 0.305-0.915 meters in length [8]. In Turkey, according to the TSE (Turkish Standards Institute), the length of the speed humps must be 3.6-3.8 meters depending on the vehicle's axle and 7.5-10 cm in height and can be placed 50-150 meters apart [10].

Speed humps are positioned along the width of the road and are preferred on larger and partially busy roads. On the other hand, speed bumps are higher and narrower; it reduces the vehicle speed much more aggressively and brings the vehicle almost to a standstill for passage. Speed humps and speed bumps are made of rubber, thermoplastic, or asphalt [7].

Speed cushions are the special design of speed humps, which are wheel cutouts for large and thick wheels that do not completely cover the road width, allowing large vehicles such as fire trucks to pass. In areas with speed cushions, large vehicles pass at driving speed while other small vehicles have to slow down [11]. But larger vehicles such as SUVs (Sport Utility Vehicles) can also easily pass by without slowing down their speed, and most drivers dangerously pass through the middle cushion of the three-piece speed cushions [12].

While traffic calming devices allow vehicles to slow down their speeds, they also cause emergency vehicles to waste time on their way to their destinations [13]. The delay experienced by ambulances reaching the emergency case or taking it to the hospital is a serious threat. Incorrectly designed, with an irregular placement and different sizes of speed humps damage the vehicles [14]. A vehicle that enters speed breakers too quickly may overturn or cause serious injury to the occupants. Especially in places lacking enough warning signs, if a high-speed vehicle approaches and brakes suddenly, the vehicle and the people in it can be seriously damaged [15]. This risk increases even more at night, in foggy, rainy weather, and for foreign drivers who have not passed that road before [16].

Emergency response vehicles have been shown to increase the time to reach their destination [17]. Such that an ambulance or a fire truck loses as much as 10 seconds while passing a standard speed bump [18]. For this reason, speed bumps are not recommended on emergency vehicle routes [7]. Passing through a speed bump can aggravate the condition, primarily by increasing injury in trauma patients. It may be difficult for health professionals in the ambulance to intervene with the patient. Also, the intervention may not be effective [19]. The ambulance driver can also change course to avoid speed humps which can cause an average of 2.5 minutes delay [20]. The delay in the passage of emergency vehicles is not only due to the slowdown of these vehicles passing through speed breakers but also the traffic jam caused by slowing down the vehicles in front of them [21].

Although there are efforts on speed breakers to facilitate the passage of emergency vehicles [22], traffic calming imposes a burden on the economy because of increasing construction costs and extra fuel consumption. In addition, the aggressive acceleration of drivers after speed breakers harms the environment by augmenting emissions [23].

Even if the vehicles pass by the rules, speed bumps can damage them and cause disturbances that require repair. They also increase the noise level in traffic by 1-5 dB due to vehicle slowing and re-accelerating [24]. Some argue that the number of lives saved thanks to traffic calming programs is less than the number of deaths in sudden cardiac disorders due to the delay caused in the emergency response time [25].

There are speed humps in different numbers and designs in different parts of each country. In the present study, we wanted to show how much time a single speed hump and two speed humps close to each other can waste ambulances and determine the queue delay time of ambulances passing through speed humps. Thus, someone who adapts this study to his region can easily calculate how much time the ambulances can lose on which route.

## Materials and methods

In the study, the width and height of 44 asphalt speed humps were measured by randomly choosing one of the three routes connecting each neighborhood to the main street in five different neighborhoods (five of the 15 routes in total). There is no speed hump on the main street. The area with a single speed hump on the route we chose from the first neighborhood was determined as the first region for the study. The area with two speed humps close to each other on the route selected from the second neighborhood was determined as the second region. In addition, a route with nine thermoplastic speed humps was chosen in a sixth district out of these five. The area with two thermoplastic speed humps on this route was determined as the third region for the study. Since all thermoplastic speed humps were the same, only one size was measured. The average speed hump number of ambulances for each route to reach the main street was 8.8 (44+9=53, 53/6=8.8).

To the Republic of Turkey's Ministry of Health quality rules, the maximum time for the ambulance to take the case to the hospital in the city after the call is received is 10 minutes. Before speed humps were made, the average time to reach the test sites from the stations where the ambulances were located was 2-3 minutes, and the average time to reach the main street from the route where the tests were carried out was 2 minutes. The average time for ambulances to reach the hospital after reaching the main street is 3 minutes.

Three types of ambulances (Van, Multiple Victim Assistance, Bariatric) and vehicles (Truck, Lorry, Van) passed in a controlled manner through speed humps at different speeds in three streets of Adıyaman province of Turkey. Ambulances and vehicles slowed to 15 km/h while passing the speed hump for safe passage. Passing and lost times were calculated with the help of a stopwatch (Catiga CG-503; Catiga Electronics Company, Hong Kong) and a global positioning system (GPS) speedometer (Vjoycar smart speedometer; Vjoy Car Electronics Limited, China).

**Figure 1:**
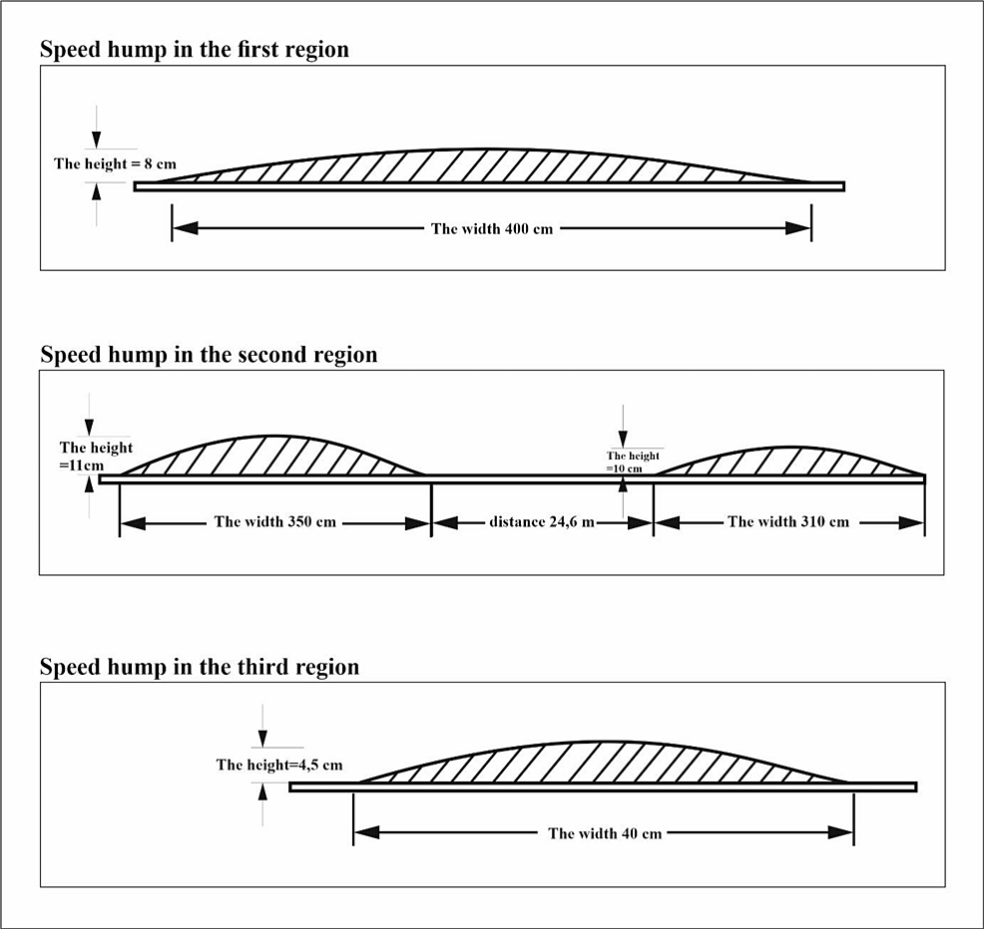
Types of the speed humps

The study was carried out by identifying the non-standard five speed humps in three streets of three regions. Speed humps, in the first and the second regions, were made of asphalt, and thermoplastic in the third region. There was one speed hump in the first region, and it was made of asphalt. There were two speed humps in the second region, and both of them were asphalt. There were two thermoplastic speed humps in the third region. Sizes of speed humps were measured with the help of meters and rulers. Since it would be more appropriate to specify the length of the bump in the latitudinal part of the road, the sizes of the speed bumps are determined as width x height instead of length x height. The width of the speed hump in the first region was 400 cm and its height was 8 cm. In the second region, there were two humps located on the same road. This region has been selected for the effect of speed humps in non-standard sizes and close to each other on time delay. The first speed hump of the second region was 350 cm wide and 11 cm high, the width of the second hump was 310 cm, its height was 10 cm, and the distance between them was 24.6 m. In the third region, there were two thermoplastic speed humps by the standards of 40 cm in width and 4.5 cm in height (Figure 1).

During the study, three types of ambulances and three types of vehicles (truck, lorry, van) were tested (Table 1). All measurements were made before noon in a rainless, windless, and fog-free environment with clear visibility for drivers. 

**Table 1:** Technical specifications of ambulances and vehicles

	Brand and Model Year	The Weight (kg)	Sizes (Length x Width x Height) (mm)
Van Ambulance	Ford Transit 2020 Model	3420	5950 x 2100 x 2900
Multiple Victim Assistance Ambulance	Wolkswagen Crafter 2019 Model	5000	7190 x 2225 x 3750
Bariatric Ambulance	Wolkswagen Crafter 2010 Model	5000	6100 x 2170 x 3750
Lorry	Ford Cargo 2021 Model	11627	8590x2540x3235
Truck	Ford Cargo 2015 Model	8063	15030x2489x3980
Van	Isuzu NPR Van 2010 Model	3540	6984x2122x2275

Vehicles passed in the first and third regions but did not pass in the second region because of traffic safety. The passing times were determined (with the help of a stopwatch) by calculating the time from the moment the ambulances and vehicles started to brake for the speed hump until they reached their pre-brake speed after passing the speed hump. Passing distances were determined (with the help of a GPS speedometer) the distance from the point at which the rear brake light lights up until the ambulances and vehicles reached their pre-brake speed after passing the speed hump. In the absence of speed humps, passing times for these distances were calculated with the speed equation formula (t = x / V). Differences in passing times in the absence and the presence of speed humps were lost times (delay times) for ambulances and vehicles. Lost times for the 50 km/h vehicles driving in front of the van ambulance were also queue delay times of the van ambulance.

In the first region, trials were conducted individually for each ambulance at pre-brake speeds of 50, 60, and 70 km/h. Also, the braking distance occurred when the van ambulance was driving behind a truck, lorry, or van, and the distance to reach the pre-brake speed again after the speed hump and total times were determined. The speed limit was set at 50 km/h so that heavy tonnage vehicles did not jeopardize traffic safety and did not include any negativity. It was tried to determine how much time the van ambulance lost when it was behind a truck, lorry, and van passing over a speed hump. Due to the heavy traffic in the region and the narrow road, the van ambulance cannot overtake the heavy tonnage vehicles in front of it. For this reason, the time lost by the vehicles while passing through the speed hump was considered the queue delay time for the van ambulance. The characteristics of the ambulances and vehicles driving in front of the van ambulance are shown in Table 1. Passing and lost time at three different speeds passing through the speed hump was calculated with the speed equation formula (t = x / V).

In the second region, trials were conducted one by one for each ambulance at speeds of 50 and 60 km/h. Due to the road situation and traffic density, no trials were carried out with a truck, a lorry, or a van in order not to experience any negativity. Passing and lost time at different speeds passing through speed breakers on the same road was calculated with the speed equation formula (t=x / V).

In the third region, trials were conducted one by one for each ambulance at speeds of 50, 60, and 70 km/h. Due to the road situation and traffic density, no trials were carried out with a truck, a lorry, or a van at a speed of 70 km/h in order not to experience any negativity.

There were no medical personnel or patients in the ambulances during the study. Since the study does not include a questionnaire, clinical or experimental study, there is no need for an ethics committee decision. Using ambulance permission was taken from the Local Health Authority (October 7, 2022/00175446238). Not to endanger traffic safety during the study, all precautions were admitted by the traffic police department.

## Results

Except for the three regions we studied, random measurements were made in various parts of the city. Only seven (15.9%) of the 44 speed breakers were found to comply with the standards (Table 2).

**Table 2:** Speed breakers measured throughout the province

Speed-Braking Humps	Type	The Width (m)	The Height (cm)		Speed-Braking Humps	Type	The Width (m)	The Height (cm)
1	Speed Hump	4.10	9.00		23	Speed Hump	3.50	6.60
2	Speed Hump	3.50	11.00		24	Speed Hump	3.60	8.60
3	Speed Hump	3.10	10.00		25	Speed Hump	3.50	7.20
4	Speed Hump	3.80	8.00		26	Speed Hump	3.30	6.40
5	Speed Hump	3.80	7.00		27	Speed Hump	3.50	12.50
6	Speed Hump	3.60	9.20		28	Speed Hump	3.80	13.40
7	Speed Hump	3.30	5.20		29	Speed Hump	3.50	8.00
8	Speed Hump	3.20	6.00		30	Speed Hump	3.50	12.00
9	Speed Hump	3.80	8.20		31	Speed Hump	3.60	9.20
10	Speed Hump	3.95	8.60		32	Speed Hump	4.00	9.00
11	Speed Hump	3.40	8.70		33	Speed Hump	3.40	7.50
12	Speed Hump	4.10	6.50		34	Speed Hump	3.40	7.50
13	Speed Hump	4.50	6.00		35	Speed Hump	1.70	12.40
14	Speed Hump	4.00	9.00		36	Speed Hump	1.60	8.70
15	Speed Hump	4.20	7.30		37	Speed Hump	1.60	11.00
16	Speed Hump	4.20	8.10		38	Speed Hump	1.10	11.50
17	Speed Hump	4.10	11.00		39	Speed Hump	1.50	8.60
18	Speed Hump	4.20	6.20		40	Speed Hump	1.00	8.70
19	Speed Hump	3.60	5.00		41	Speed Hump	1.60	0.70
20	Speed Hump	3.80	8.50		42	Speed Hump	1.00	10.50
21	Speed Hump	3.60	6.50		43	Speed Hump	1.20	7.50
22	Speed Hump	3.80	7.80		44	Speed Hump	1.10	9.50

In the first region

The lost time for the van ambulance with a speed of 50 km/h was 5.74 seconds, 8.96 seconds for the multiple victim assistance (MVA) ambulance, and 5.12 seconds for the bariatric ambulance. The lost time for the van ambulance with a speed of 60 km/h was 7.94 seconds, 9.84 seconds for the MVA ambulance, and 7.22 seconds for the bariatric ambulance. The lost time for the ambulance with a speed of 70 km/h was 8.41 seconds, 10.14 seconds for the MVA ambulance, and 9.56 seconds for the bariatric ambulance (Table 3). The total minimum lost time will be 50.51, 78.85, and 45.06 seconds for an average of 8.8 humps on the first route. The maximum lost time will be 74.00, 89.23, and 84.13 seconds.

**Table 3:** Data for the ambulances studied in the first region

	Braking Distance (meters)			Distance to Pre-brake Speed Again (meters)			Passing Time (seconds)			Lost Time (seconds)		
Velocity	50 km/h	60 km/h	70 km/h	50 km/h	60 km/h	70 km/h	50 km/h	60 km/h	70 km/h	50 km/h	60 km/h	70 km/h
Van Ambulance	40	42	60	72	112	154	13.81	17.18	17.18	5.74	7.94	8.41
Multiple Victim Assistance Ambulance	43	45	87	82	132	166	17.96	20.46	20.46	8.96	9.84	10.14
Bariatric Ambulance	74	78	93	78	128	161	16.06	19.48	19.48	5.12	7.22	9.56

While a truck in front of the van ambulance had a speed of 50 km/h in the first zone, the lost time was 54.96 seconds, the lost time with a lorry was 42.81 seconds, and 7.02 seconds with a van (Table 4). The maximum queue delay time for 8.8 humps will be 483.65, 376.73, and 61.78 seconds.

**Table 4:** Data for the vehicles (V= 50 km/h) studied in the first region

	Braking Distance (meters)	Distance to Pre-brake Speed Again (meters)	Total Time (seconds)	Lost Time^*^ (seconds)
Truck	112	114	71.23	54.96
Lorry	96	102	57.16	42.81
Van	45	94	17.10	7.02

In the second region

The lost time for the van ambulance with a speed of 50 km/h was 9.39 seconds, 13.2 seconds for the MVA ambulance, and 11.97 seconds for the bariatric ambulance. The lost time for the van ambulance with a speed of 60 km/h was 9.94 seconds, 16.32 seconds for the MVA ambulance, and 14.49 seconds for the bariatric ambulance (Table 5). For an average of 8.8 (4.4 doubles) speed humps on the second route, the total minimum lost time will be 41.32, 58.08, and 52.67 seconds. The maximum lost time will be 43.74, 71.81, and 63.76 seconds.

**Table 5:** Data for the ambulances studied in the first region

	Braking Distance (meters)		Distance to Pre-brake Speed Again (meters)		Passing Time ( seconds)		Lost Time ( seconds)	
	V 50 km/h	V 60 km/h	V 50 km/h	V 60 km/h	V 50 km/h	V 60 km/h	V 50 km/h	V 60 km/h
Van Ambulance	56	54	82.6	101.6	18.65	20.64	9.39	9.94
Multiple Victim Assistance Ambulance	50	58	98.6	117.6	22.53	26.85	13.2	16.32
Bariatric Ambulance	62	68	91.6	108	23.02	25.05	11.97	14.49

In the third region

In the measurements made in the third region, all types of ambulances at 50, 60, and 70 km/h could pass comfortably without reducing their speed during the transition from thermoplastic speed humps. Also, the other vehicles (the truck, the lorry, and the van) with 50 km/h and 60 km/h speeds did not brake and passed thermoplastic speed humps without slowing down. Therefore, delay time was zero in the thermoplastic humps in the third region.

## Discussion

In the present study, only 15.9% of randomly looked-at speed humps were by the standards. Since only a few speed humps comply with the standard, non-standard speed humps were tested in our study to determine the time loss of ambulances in real life. Speed breakers that do not comply with the standards cause serious damage to both vehicles and their occupants [14]. Therefore, they should be built by standards and checked strictly.

In a study, speed humps caused a delay of up to 9.4 seconds. But queue delay time because of the traffic jam was not considered. [18]. In an observational study, the queue delay time in speed humps was 2.02 to 3.45 seconds [8]. The present study is experimental instead of observational. The passing times of ambulances and vehicles through speed humps were measured for delay and queue delay times of ambulances. Compared to Atkins and Coleman's study in 1997 [18], more different delay times were recorded. The delay time was a maximum of 8.41 seconds with the van ambulance and 10.14 seconds with the MVA ambulance in the first region and was 9.94 and 16.32 seconds in the second region with double speed humps of different sizes. Queue delay times were much higher than Al-Omari and Al-Masaeid's observational study [8]. However, in the present study, heavy tonnage vehicles, which cause the most queuing in traffic, were used to show how much of a waste of time speed humps can cause for ambulances. When passing through a speed hump, the queue delay time is 7.02 seconds for a van and 54.96 seconds for a truck.

Ambulances experience a serious loss of time while passing speed humps, depending on their types. Moreover, when there are heavy tonnage vehicles in front of them, the queue delay times of the ambulances are very high. As the speed of ambulances and vehicles increased, the braking distance and the time to reach the pre-brake speed increased, so the lost time also increased. Hence, more time is lost when the ambulance needs to go faster. This delay can threaten the life of patients in emergencies.

In the present study, in order not to waste time for emergency transitions, ambulances and other vehicles passed through the speed humps at 15 km/h tolerable speed at a speed hump, safe transition. However, in a study, a fire brigade with a fully equipped vehicle passes a 10-cm high-speed bump at 8 km/h, and it has no negative impact on those in the cabin. Patients may be mildly affected. No objects jump into the air. When it passes at 16 km/h speed, a low level of undesirable effects can occur in patients with spinal damage. Unsecured objects can jump into the air [3]. Although it is appropriate not to waste time for ambulances and vehicles to pass the speed hump at a tolerable speed of 15 km/h for safe passage, it is much more appropriate to have 8 km/h for the vehicle and cabin occupants to be unaffected. Because in spinal damage and other trauma patients, every concussion in the ambulance can worsen the patient's condition. So, when the deceleration speed of ambulances and vehicles passing through the speed hump was 8 km/h instead of 15 km/h, the vehicle transit times would be much longer.

For pedestrian safety, instead of speed bumps and speed humps, roundabouts, alternative traffic lanes, chokers, speed signs, stop lights, and speed cameras can be used [3,12]. However, if speed breakers are thought to be the most effective way to pedestrian safety, speed cushions can be even more effective and convenient. Although speed humps and speed cushions of the same height and length have a similar effect on speed control, some argue that speed cushions do not increase the response time because they do not cause a decrease in the speed of fire trucks [26]. However, in some countries, such as Turkey, it may not be possible to design a speed cushion that all emergency vehicles can pass through, as the size of ambulances and fire trucks are different. Instead, it may be more convenient to use chokers or speed kidneys, a new generation of speed lumps consisting of three speed clusters. The speed kidney has a curvilinear shape that allows trucks and emergency vehicles, such as fire trucks, to pass without slowing down [27].

As a strength of the present study, in the literature, there is no study conducted with different speeds, different types and sizes of speed humps, and different ambulance types together. Moreover, previous studies on queue delay were observational; on the contrary, the present study is an original study based on measurement data. The model years of ambulances and vehicles differed from each other, which is the study's limitation.

## Conclusions

In the present study, trials with speed bumps in three different regions showed a significant delay in ambulances passing speed humps, depending on the type of ambulance and the type of vehicles in front of it. Ambulances also suffered a queue delay time because of the vehicles in front of them. This delay does not only affect the time for the ambulance to reach the patients but also the patients’ reach time to the hospital. As the speed of ambulances increased, the lost time also increased. So, more time is lost when the ambulance needs to go faster. Using something other than speed breakers or using them in limited places according to the standards may be beneficial. Portable speed breakers can also be the solution in very mandatory situations. The thermoplastic speed breakers do not affect the passing time for ambulances and other vehicles, so their effectivity of decreasing the vehicles’ speed for the safety of pedestrians is debatable.
